# Randomized crossover comparison of two teriparatide self-injection regimens for primary osteoporosis: Interim report (end of 52-week treatment) of the Japanese Osteoporosis Intervention Trial 06 (JOINT-06)

**DOI:** 10.1007/s00774-025-01586-y

**Published:** 2025-02-18

**Authors:** Satoshi Soen, Yukari Uemura, Shiro Tanaka, Yasuhiro Takeuchi, Naoto Endo, Junichi Takada, Satoshi Ikeda, Jun Iwamoto, Nobukazu Okimoto, Sakae Tanaka

**Affiliations:** 1 Soen Orthopaedics, Osteoporosis and Rheumatology Clinic, 7-12-60 Okamoto, Higashinada-Ku, Kobe, Hyogo 658-0071 Japan; 2 Biostatistics Section, Department of Data Science, Center for Clinical Sciences, National Center for Global Health and Medicine, Toyama, Shinjuku-Ku, Tokyo, Japan; 3 Department of Clinical Biostatistics, Graduate School of Medicine, Kyoto University, Yoshida Konoe-Cho Sakyo-Ku, Kyoto, Japan; 4 Toranomon Hospital Endocrine Center, Toranomon, Minato-Ku, Tokyo Japan; 5 Okinaka Memorial Institute for Medical Research, Toranomon, Minato-Ku, Tokyo Japan; 6 Department of Orthopedic Surgery, Saiseikai Niigata Kenoh Kikan Hospital, Kamisugoro, Sanjo, Niigata, Japan; 7 Osteoporosis Center, Sapporo Maruyama Orthopaedic Hospital, Chuo-Ku, Sapporo, Hokkaido Japan; 8 Department of Orthopaedic Surgery, Ken-Ai Memorial Hospital, Oaza Kimori, Onga-Machi, Onga, Fukuoka Japan; 9 Bone and Joint Disease Center, Keiyu Orthopaedic Hospital, Akoudacho, Tatebayashi, Gunma Japan; 10 Okimoto Clinic, Kubi, Yutakamachi, Kure, Hiroshima Japan; 11 Department of Orthopaedic Surgery, Faculty of Medicine, University of Tokyo, Hongo, Bunkyo-Ku, Tokyo Japan

**Keywords:** Osteoporosis, Self-injection regimen, Patient satisfaction, Randomized controlled trial, Teriparatide

## Abstract

**Introduction:**

Patient satisfaction with two teriparatide (TPTD) self-injection regimens [once-daily (1/D)-TPTD and twice-weekly (2/W)-TPTD] was compared in a randomized crossover study involving patients with osteoporosis at high fracture risk.

**Materials and Methods:**

Questionnaires evaluated overall satisfaction, satisfaction with treatment effectiveness, satisfaction with utility of the self-injection device, and preference for a particular injection regimen after crossover. Quality of life (QOL), visual analogue scale pain scores, and bone mineral density (BMD) were also analyzed. Safety was evaluated based on the incidence and severity of adverse events (AEs).

**Results:**

The 1/D-TPTD and 2/W-TPTD groups comprised 180 (mean age: 75.9 ± 7.3 years) and 178 (75.4 ± 6.9 years) patients, respectively. After 26 weeks of treatment, the injection regimens were switched and treatment continued for another 26 weeks. Significantly higher persistence was observed in the 1/D-TPTD to 2/W-TPTD group (p = 0.032). No significant between-group differences in overall satisfaction scores or satisfaction with treatment were observed. Satisfaction with the utility of the injection device was significantly higher with the 2/W-TPTD regimen (p < 0.05); this regimen was preferred by 69.4% of patients after crossover (p < 0.001). A significant increase in BMD from baseline was observed at the lumbar vertebrae in both groups and at the hip area in the 1/D-TPTD to 2/W-TPTD group at 52 weeks (p < 0.05). Significant improvement in the QOL score was observed in both groups (p < 0.05). No serious AEs were reported.

**Conclusion:**

Continuation of this study will further clarify patient satisfaction, treatment effects, and tolerability.

**Supplementary Information:**

The online version contains supplementary material available at 10.1007/s00774-025-01586-y.

## Introduction

Osteoporosis affects more than 200 million women and causes 8.9 million fractures per year worldwide. Osteoporotic fractures are associated with high mortality and disability rates and high medical costs [1]. Early diagnosis and prompt initiation of treatment are important for effective management of bone fractures. Sustained treatment is also necessary to stimulate new bone formation in patients of advanced age. To ensure sustained treatment, patients should be satisfied with their treatment or well-educated about its significance through continuous follow-up by medical personnel [2–6]. Thus, evaluation of patient satisfaction has been an important endpoint in recent clinical trials evaluating agents for the treatment of osteoporosis [7–9]. Treatment compliance has been shown to correlate with patient satisfaction [10, 11].

Several different treatment formulations of teriparatide (TPTD) are currently available in Japan for the treatment of osteoporosis [2, 4, 6, 12]. We conducted a randomized crossover comparison study (26 weeks for each treatment period) to evaluate patient satisfaction with two self-injection regimens: daily injection of TPTD (1/D-TPTD) and twice-weekly injection of TPTD (2/W-TPTD) [13]. The self-injection device used to deliver the 2/W formulation has an invisible needle that does not require replacement, unlike the 1/D device. We found that both overall patient satisfaction and patient satisfaction with treatment did not differ significantly between the two regimens. However, patient satisfaction with the utility of the injection regimen tended to favor the 2/W-TPTD regimen, suggesting that factors such as ease of preparation, handling, and usability of the injection device; frequency of injection; and pain at the injection site may affect patient satisfaction and contribute to treatment persistence.

## Purpose

We herein report the results of the second period of the above-mentioned crossover study in combination with the first-period data. These results show significantly higher satisfaction with the utility of the 2/W-TPTD injection regimen over the 1/D-TPTD regimen as well as greater preference for the 2/W-TPTD regimen during the 52-week free-choice period.

## Materials and methods

### Study patients

This multicenter crossover study (jRCTs031210187) was conducted at 39 centers in Japan from July 2021 to September 2023. Postmenopausal women who had primary osteoporosis [14], were aged ≥ 60 years, and had a high fracture risk were eligible if they satisfied any of the following inclusion criteria: bone mineral density (BMD) of < 60% of the young adult mean or < − 3.3 standard deviation (SD), ≥ 2 vertebral fractures (assessed by a semiquantitative method [15]) between the fourth thoracic vertebra (Th4) and fourth lumbar vertebra (L4), a grade 3 vertebral fracture, ≥ 1 vertebral fracture at Th4–L4 and BMD of ≤ − 2.5 SD of the young adult mean, or a history of hip fracture. Patients with secondary osteoporosis, with bone loss due to diseases other than osteoporosis, receiving medications that may modify the effect of TPTD, with experience using an auto-injection device, or who had previously received TPTD treatment were excluded. Full details are provided in our previous report [13].

### Study design

Eligible patients were randomized into two groups. One group received a once-daily dose of TPTD (20 µg self-injection) (1/D-TPTD group) and the other received a twice-weekly dose of TPTD (28.2 µg self-injection) (2/W-TPTD group) for 26 weeks (the first quarter of the 104-week study period). The dosing regimen was then switched, and treatment was continued for another 26 weeks (one half of the total study period). After completion of the 52-week crossover period, the patients were allowed to receive either a daily dose or twice-weekly dosing according to their preference, and the treatment was continued for another 52 weeks (until the end of the study). All patients received daily vitamin D supplementation (25 µg/day) throughout the study period.

### Endpoints

Patient satisfaction was investigated using the Patient Satisfaction Questionnaire [13]. This questionnaire consists of one question regarding overall satisfaction (asked at 26 and 52 weeks), two questions regarding the effectiveness of treatment (asked at 26 and 52 weeks), and 12 questions regarding the utility of the injection device (asked at 2, 4, 13, and 26 weeks), with ease of use rated from difficult to easy on a 3-point or 6-point scale. The primary endpoint of the study was the degree of overall satisfaction at 26 weeks. The secondary endpoints were (1) overall patient satisfaction at 52 and 104 weeks, (2) patient satisfaction with treatment at 26, 52, and 104 weeks, (3) the time course of patient satisfaction with device utility (12 questions), (4) preference for a particular injection regimen when the patient was allowed to choose at 52 weeks, (5) adherence to the treatment, and (6) efficacy of the treatment. Adherence was judged to be good if the overall self-injection rate was ≥ 80%, the self-injection rate was ≥ 50% during the final 4 weeks, and the attendance at the final visit was within the predetermined time limit. Treatment efficacy was judged by the number of incident fractures, change in BMD, quality of life (QOL) according to the EuroQoL-5 Dimension scale, pain according to a visual analogue scale (VAS), and parameters of bone structure analysis. Dual-energy X-ray absorptiometry was used to measure BMD of first lumbar vertebra (L1)–L4 or second lumbar vertebra (L2)–L4, the femoral neck, and the total hip. The safety of treatment was evaluated based on the incidence, type, and severity of adverse events (AEs).

### Statistical analyses

Assuming a score difference of 0.7 between the two groups and an SD of 2.0, a sample size of 180 patients was required to achieve > 90% power with a two-sided alpha error of 5%. Therefore, accounting for an expected dropout rate of 10%, the target sample size was set at 200 patients. Endpoints were analyzed in the full analysis set. All data are presented as mean ± SD or number and percentage. The differences in baseline characteristics between the 1/D-TPTD and 2/W-TPTD groups were evaluated by the t-test for continuous variables and Fisher’s χ^2^ test for categorical variables. The mean and SD of the patient satisfaction scores were determined, and differences between groups were evaluated using the t-test. The patients’ treatment preferences at 52 weeks and the incidence of clinical fracture were evaluated by the χ^2^ test and McNemar’s test, respectively. The paired t-test was used to assess time-dependent changes in QOL scores, VAS scores, and anthropometric parameters from baseline. To evaluate the time-dependent dropout rate, Kaplan–Meier plots were used and evaluated with the log-rank test. Differences with p values of < 0.05 were considered statistically significant. All statistical analyses were performed using SAS software version 9.4 (SAS Institute Inc., Cary, NC, USA).

### Ethics and consent

All procedures performed in studies involving human participants were in accordance with the ethical standards of the institutional and/or national research committee and with the 1964 Helsinki declaration and its later amendments or comparable ethical standards. The study protocol was approved by the respective clinical research review boards. Written informed consent was obtained from all individual participants included in the study.

## Results

In total, 358 patients (180 in the 1/D-TPTD group and 178 in the 2/W-TPTD group) were enrolled in the 26-week initial phase of the crossover treatment. At the beginning of the 27th week, the treatment regimen was switched (146 patients from 1/D-TPTD to 2/W-TPTD and 130 patients from 2/W-TPTD to 1/D-TPTD), and the study was continued for another 26 weeks.

### Patient characteristics

The patients’ baseline characteristics are shown in Online Resource 1. The patients’ mean age at baseline was 75.9 ± 7.3 years in the 1/D-TPTD to 2/W-TPTD group and 75.4 ± 6.9 years in the 2/W-TPTD to 1/D-TPTD group, and no significant difference was observed between the groups (p = 0.511). The T-score of lumbar vertebral BMD (L2–L4) was − 2.27 ± 1.80 in the 1/D-TPTD to 2/W-TPTD group and − 2.48 ± 1.29 in the 2/W-TPTD to 1/D-TPTD group (p = 0.284). No difference in femoral neck BMD or total hip BMD was observed between the groups. No significant difference was observed between the groups.

### Treatment persistence

**Fig. 1:**
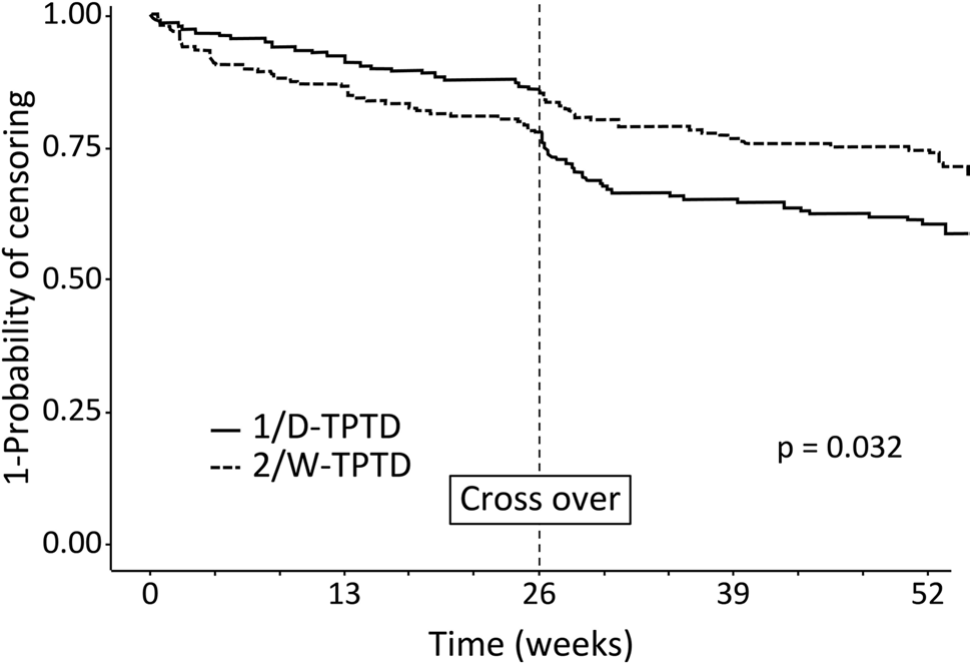
Kaplan–Meier plot of time-dependent dropout rate. 1/D-TPTD, daily injection of teriparatide; 2/W-TPTD, twice-weekly injection of teriparatide

The treatment persistence rates at 26 and 52 weeks were 82.2% and 83.6% in the 1/D-TPTD to 2/W-TPTD group (p = 0.312) and 78.1% and 79.2% in the 2/W-TPTD to 1/D-TPTD group (p = 0.322). No statistically significant difference in persistence was observed between the two dosing regimens in each 26-week period before and after crossover. The time-dependent dropout rate during the total 52 weeks is shown in Fig. 1. Significantly higher persistence was observed in the 1/D-TPTD to 2/W-TPTD group (p = 0.032).

### Endpoints

The degree of overall patient satisfaction (the primary endpoint of the study) and the degree of satisfaction with the effectiveness of treatment are summarized in Table 1. No statistically significant difference was observed between the dosing regimens at either 26 or 52 weeks. Table 2 summarizes the patients’ satisfaction with the utility of the injection device. The scores for satisfaction with the utility of the device in response to Q1, Q2, Q3, Q4, Q6, and Q9 were significantly higher at 52 weeks than at 26 weeks in the 1/D-TPTD to 2/W-TPTD group. Conversely, the scores for satisfaction with the utility of the device in response to Q4, Q6, and Q9 were significantly lower at 52 weeks than at 26 weeks in the 2/W-TPTD to 1/D-TPTD group. These results indicate that with the 2/W-TPTD regimen, the device is easier to prepare and handle, injections are easier to successfully perform, less frequent injection is necessary, and less pain is experienced at the injection site.

**Table 1:** Patients’ overall satisfaction and patients’ satisfaction with treatment

Items		Question	TPTD		26 weeks	52 weeks	p
			Initiation	Crossover			
Overall satisfaction		How would you rate your satisfaction with this teriparatide preparation? Please rate your satisfaction on a scale of 0 to 5 (0: dissatisfied to 5: satisfied)	1/D	2/W	3.5 ± 1.3	3.6 ± 1.1	0.506
			2/W	1/D	3.4 ± 1.2	3.4 ± 1.1	0.942
Satisfaction with treatment	Q1	Do you feel positive effects of the teriparatide preparation being used? Please rate the effects on a scale of 0 to 5 (0: no to 5: yes)	1/D	2/W	2.7 ± 1.7	3.0 ± 1.6	0.187
			2/W	1/D	2.6 ± 1.8	2.8 ± 1.6	0.062
	Q2	Do you feel improvement of lower or upper back pain with the teriparatide preparation being used? Please rate the improvement on a scale of 0 to 5 (0: no to 5: yes)	1/D	2/W	2.8 ± 1.8	3.0 ± 1.6	0.129
			2/W	1/D	2.5 ± 1.8	2.6 ± 1.7	0.072

Data are presented as mean ± standard deviation

*TPTD* teriparatide; *1/D* once daily; *2/W* twice weekly

Differences between the groups were evaluated by the paired t-test

**Table 2:** Patient-reported satisfaction with self-injection device utility

	Question	TPTD		26 weeks	52 weeks	p
		Initiation	Crossover			
Q1	How do you feel about the preparation for injection? Please evaluate on a scale of 0 to 5 (0: difficult to 5: easy)	1/D	2/W	4.4 ± 1.1	4.6 ± 0.7	0.042
		2/W	1/D	4.4 ± 1.0	4.5 ± 0.9	0.586
Q2	During injection, how do you feel about the handling and usability of this injection device? (0: difficult to 5: easy)	1/D	2/W	4.4 ± 1.1	4.6 ± 0.7	0.026
		2/W	1/D	4.4 ± 1.0	4.5 ± 0.9	0.348
Q3	After injection, can you confirm whether your injection was successful? Please rate the process on a scale of 0 to 5 (0: difficult to 5: easy)	1/D	2/W	4.3 ± 1.1	4.6 ± 0.8	0.022
		2/W	1/D	4.4 ± 0.9	4.5 ± 0.8	0.274
Q4	Have you experienced failed injection? Please specify how often you failed during the past 2 weeks or 1 month by placing a check mark in the appropriate checkbox (0: none; 4: ≥ 4 times; 9: unknown)	1/D	2/W	0.3 ± 0.8	0.2 ± 0.9	0.021
		2/W	1/D	0.1 ± 0.9	0.3 ± 1.0	0.007
Q5	How long does it take for you to complete the whole injection procedure, from preparation to finish? Please specify by placing a check mark in the appropriate checkbox (1: 0–5 min; 2: 5–10 min; 3: ≥ 10 min)	1/D	2/W	1.2 ± 0.5	1.2 ± 0.5	0.371
		2/W	1/D	1.3 ± 0.6	1.2 ± 0.4	0.401
Q6	How do you feel about the frequency of injection? Please rate it on a scale 0 to 5 (0: dissatisfied to 5: satisfied)	1/D	2/W	3.8 ± 1.4	4.3 ± 1.1	0.001
		2/W	1/D	3.9 ± 1.3	3.7 ± 1.3	0.002
Q7	How do you feel about continuing self-injection? Please rate it on a scale of 0 to 5 (0: difficult to 5: easy)	1/D	2/W	4.0 ± 1.4	4.2 ± 1.2	0.181
		2/W	1/D	3.9 ± 1.2	3.9 ± 1.2	0.367
Q8	How do you feel about the safety of self-injection? Please rate it on a scale of 0 to 5 (0: unsafe to 5: safe)	1/D	2/W	4.4 ± 1.0	4.4 ± 1.0	0.668
		2/W	1/D	4.5 ± 0.8	4.4 ± 0.8	0.278
Q9	Do you have pain at the injection site? Please rate it on a scale of 0 to 5 (0: yes to 5: no)	1/D	2/W	3.7 ± 1.3	4.1 ± 1.2	0.004
		2/W	1/D	4.0 ± 1.2	3.8 ± 1.2	0.031
Q10	How do you feel about the storage of the injection set (syringe, needle, etc.)? Please rate your feeling on a scale of 0 to 5 (0: difficult to 5: easy)	1/D	2/W	4.6 ± 0.8	4.6 ± 0.8	0.595
		2/W	1/D	4.4 ± 0.9	4.5 ± 0.8	0.909
Q11	How do you feel about the storage and disposal of used injection sets? Please rate your feeling on a scale of 0 to 5 (0: difficult to 5: easy)	1/D	2/W	4.5 ± 0.9	4.5 ± 0.9	0.804
		2/W	1/D	4.5 ± 0.8	4.5 ± 0.8	0.307
Q12	How do you feel about self-injection? Please rate your feeling on a scale of 0 to 5 (0: difficult to 5: easy)	1/D	2/W	4.2 ± 1.2	4.3 ± 1.2	0.933
		2/W	1/D	4.1 ± 1.2	4.1 ± 1.1	0.128

Data are presented as mean ± standard deviation

*TPTD* teriparatide; *1/D* once daily; 2/W, twice weekly

Differences between the groups were evaluated by the paired t-test

### Preference for injection regimen

When the patients were allowed to choose the treatment regimen at 52 weeks, 69.4% of the patients chose the 2/W injection regimen (p < 0.001). The reasons for the patients’ preferences are shown in Table 3. The most common reasons for choosing the 2/W injection regimen were satisfaction with the frequency of injections, ease of preparing the device for injections, ease of operating the syringe, ease of learning how to use the device, and ease of injection. The rate of preference for the 2/W injection regimen by crossover group was 86.9% in the 1/D-TPTD to 2/W-TPTD group and 47.1% in the 2/W-TPTD to 1/D-TPTD group (p < 0.001).

**Table 3:** Reasons for drug regimen preferences during free-choice study period

Reason	1/D-TPTD		2/W-TPTD	
Easy to learn how to use	27	(38.0)	93	(57.8)
Easy to prepare for injections	27	(38.0)	100	(62.1)
Easy to operate syringe	26	(36.6)	99	(61.5)
Easy to check after injection	21	(29.6)	73	(45.3)
Less prone to injection errors	21	(29.6)	76	(47.2)
Short injection time	24	(33.8)	71	(44.1)
Satisfied with the frequency of injections	19	(26.8)	119	(73.9)
Easy to continue injecting	28	(39.4)	75	(46.6)
Feels safe to inject	12	(16.9)	54	(33.5)
Less pain when injecting	24	(33.8)	59	(36.6)
Easy to store before use	24	(33.8)	57	(35.4)
Easy to store and dispose of after use	22	(31.0)	59	(36.6)
Easy to inject	20	(28.2)	89	(55.3)
Feel the effect of treatment	10	(14.1)	32	(19.9)
Feel improvement in pain from treatment	9	(12.7)	24	(14.9)
Overall satisfaction	16	(22.5)	59	(36.6)
Other	26	(36.6)	3	(1.9)

Data are presented as n (%)

*1/D-TPTD* daily injection of teriparatide; *2/W-TPTD* twice-weekly injection of teriparatide

### Clinical fracture

The number and percentage of incident clinical fracture in the 1/D-TPTD to 2/W-TPTD group were 0/180 (0.0%) in the first 26 weeks and 3/146 (2.1%) in the next 26 weeks after crossover (p = 0.083). In the 2/W-TPTD to 1/D-TPTD group, 4/178 (2.2%) and 1/130 (0.8%) fractures were observed in each period (p = 0.317).

### Effects on bone mineral density

**Fig. 2:**
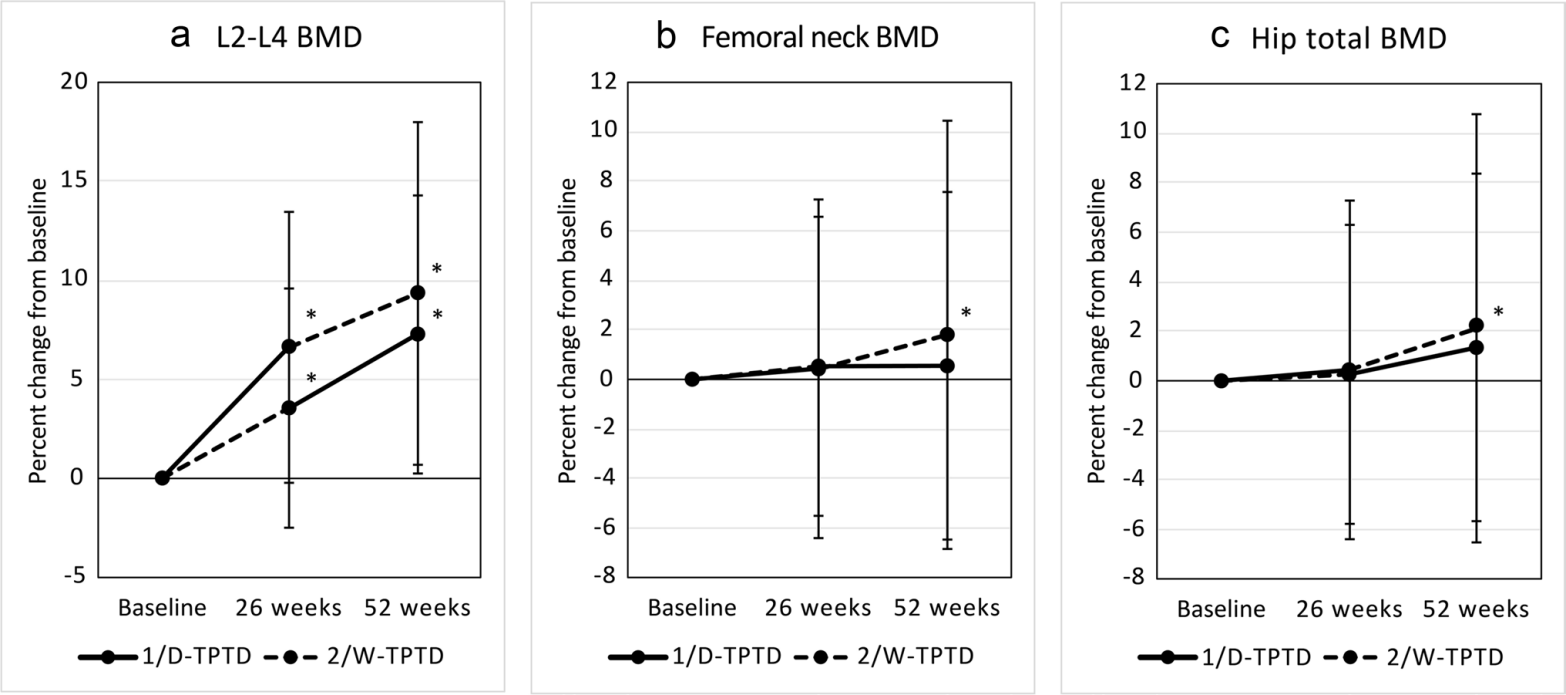
Effects of treatment on BMD of lumbar vertebrae, femoral neck, and total hip. Each point and bar indicate the mean ± standard deviation. Difference from baseline was evaluated by the t-test. *p < 0.001. *1/D-TPTD* daily injection of teriparatide; *2/W-TPTD* twice-weekly injection of teriparatide; *BMD* bone mineral density; *L2* second lumbar vertebra; *L4* fourth lumbar vertebra

Figure 2a–c shows the treatment-induced percent change from baseline in the BMD of L2–L4, the femoral neck, and the total hip. Both 1/D-TPTD to 2/W-TPTD and 2/W-TPTD to 1/D-TPTD treatment induced a time-dependent increase in L2–L4 BMD (9.3% ± 8.7% and 7.3% ± 7.0% at 52 weeks, respectively). A significant increase was observed in the femoral neck BMD (1.8% ± 8.6%) and total hip BMD (2.1% ± 6.7%) at 52 weeks only in the 1/D-TPTD to 2/W-TPTD group.

The effects of treatment on QOL, pain, height, and body weight are summarized in Table 4. At 52 weeks, the EuroQoL-5 Dimension utility score was significantly improved in both the 1/D-TPTD to 2/W-TPTD and 2/W-TPTD to 1/D-TPTD groups (p = 0.003 and p = 0.029), the pain VAS score was significantly lower in the 1/D-TPTD to 2/W-TPTD group (p = 0.002), and height was significantly lower in both treatment groups (p < 0.001 and p = 0.013, respectively). However, the difference in body weight did not reach statistical significance (p = 0.057 and p = 0.074, respectively).

**Table 4:** Effects of treatment on quality of life, pain, body height, and weight

		1/D-TPTD to 2/W-TPTD			2/W-TPTD to 1/D-TPTD		
		Mean ± SD	n	p	Mean ± SD	n	p
EQ-5D, utility score	Baseline	0.90 ± 0.21	180		0.92 ± 0.19	176	
	26 weeks	0.95 ± 0.15	153		0.98 ± 0.10	139	
	52 weeks	0.96 ± 0.13	129		0.97 ± 0.11	110	
	Absolute change	0.06 ± 0.21	129	0.003	0.04 ± 0.16	109	0.029
Pain VAS score	Baseline	31.5 ± 29.8	179		28.6 ± 29.5	174	
	26 weeks	20.6 ± 22.6	152		22.7 ± 26.0	141	
	52 weeks	21.3 ± 24.2	130		21.5 ± 27.3	104	
	Absolute change	− 8.3 ± 29.9	129	0.002	− 6.0 ± 33.0	102	0.071
Height, cm	Baseline	150.3 ± 6.1	171		150.1 ± 6.1	171	
	26 weeks	149.5 ± 6.4	133		150.3 ± 6.3	122	
	52 weeks	149.6 ± 6.2	108		150.5 ± 6.7	86	
	Absolute change	− 0.9 ± 2.2	107	< 0.001	− 0.5 ± 1.9	84	0.013
Body weight, kg	Baseline	49.6 ± 7.7	171		50.0 ± 8.8	171	
	26 weeks	49.4 ± 8.1	134		50.4 ± 9.1	123	
	52 weeks	49.6 ± 7.4	108		50.6 ± 8.8	86	
	Absolute change	− 0.5 ± 2.8	107	0.057	− 0.5 ± 2.5	84	0.074

*EQ-5D* EuroQoL-5 Dimension scale; *VAS* visual analogue scale; *1/D-TPTD* daily injection of teriparatide; *2/W-TPTD* twice-weekly injection of teriparatide; *SD* standard deviation

Differences from baseline were evaluated by the paired t-test

### Adverse events

In the period before and after the crossover, AEs that occurred during each treatment period in the 1/D-TPTD and 2/W-TPTD groups are summarized in Online Resource 2. AEs occurred in 13/310 (4.2%) patients in the 1/D-TPTD group and in 22/324 (6.8%) patients in the 2/W-TPTD group, but none of the AEs were serious.

## Discussion

This randomized multicenter crossover trial, consisting of 26 weeks for each period, compared patient satisfaction with two different TPTD formulations: 1/D-TPTD and 2/W-TPTD. At the end of the first and second periods of the crossover study, the persistence of self-injection did not differ between the 1/D-TPTD and 2/W-TPTD groups. Neither overall patient satisfaction nor treatment satisfaction differed between the two injection regimens at the end of both periods. By contrast, patient satisfaction with utility of the device differed between the two injection regimens after the end of the second period (at 52 weeks). This difference was associated with preparation for injection, handling and usability of the device, the injection success rate, the frequency of injection, and pain at the injection site. At 52 weeks, the patients in the 1/D-TPTD to 2/W-TPTD group showed significantly higher satisfaction with utility of the 2/W-TPTD regimen for the following reasons: the device was easier to prepare, handle, and use; the injection success rate was higher; less frequent injections were needed; and less pain was experienced at the injection site. By contrast, the patients in the 2/W-TPTD to 1/D-TPTD group showed significantly lower satisfaction with the 1/D-TPTD regimen for the following reasons: the rate of successful injection was lower, frequent injections were needed, and more pain was experienced at the injection site. At the end of the crossover, the patients were allowed to choose either one of the two injection regimens, and nearly 70% of patients chose the 2/W-TPTD injection regimen with statistical significance. The reasons for this preference were the low frequency of injections, ease of preparing for injections, ease of operating the syringe, ease of learning how to use the device, and ease of injection.

Although there was no difference in the persistence rate at 26 weeks before and after crossover, a significantly higher persistence rate was observed in the 1/D-TPTD to 2/W-TPTD group throughout the entire 52 weeks. Differences in patient satisfaction with the device utility may have influenced the treatment discontinuation rate, and switching from 2/W-TPTD to 1/D-TPTD may have reduced the persistence rate.

It is not practical for patients to switch between two TPTD formulations in a clinical setting and experience the difference. Therefore, physicians considering prescribing TPTDs need to explain to patients not only the difference in dosing frequency, but also the difference in utility of device including preparation requirements with reference to the results of this study. Prescribing a dosage form that satisfies patients may be important in preventing treatment discontinuation.

Notably, patients both in the 1/D-TPTD to 2/W-TPTD group and 2/W-TPTD to 1/D-TPTD group exhibited a statistically significant increase in L2–L4 BMD from baseline at both 26 and 52 weeks, and the effects were time-dependent. The femoral neck and total hip BMDs also significantly increased from baseline at 52 weeks in the 1/D-TPTD to 2/W-TPTD group, but not in the 2/W-TPTD to 1/D-TPTD group. This difference in changes of BMD may be explained by the different effects of the two dosing regimens of TPTD on changes in bone turnover markers. Administration of 1/D-TPTD reportedly increases both bone formation and resorption [16], whereas 2/W-TPTD increases bone formation and decreases bone resorption [12, 17]. The increase in bone resorption by administration of 1/D-TPTD may be suppressed by switching to 2/W-TPTD, leading to a greater increase in BMD. The QOL score significantly improved in both groups. The VAS pain score also tended to improve, but statistical significance was observed only in the 1/D-TPTD to 2/W-TPTD group. Body height significantly decreased in both groups. The frequency of AEs tended to be lower than the results of the respective development trials [12, 16]. These differences may have been due to differences in the frequency of visits to the hospital and periodic vital examinations.

A limitation of this study is that the data were obtained from a randomized, allocation-controlled study and may differ from the data obtained in actual clinical situations. However, several significant differences were observed between the two dosing regimens, and there was a clear difference in the final treatment preference. This study is still in progress and will continue for another 52 weeks. It may be possible to report more definitive conclusions regarding persistence based on differences in patient preferences, patient satisfaction, and efficacy of the two injection regimens.

## Conclusion

This randomized, crossover comparison study demonstrated that overall patient satisfaction and satisfaction with treatment were not different between the 1/D-TPTD and 2/W-TPTD self-injection regimens. However, patient satisfaction with the utility of the injection device was higher in the 2/W-TPTD group, and 69.4% of patients preferred the 2/W-TPTD injection regimen over the 1/D-TPTD regimen when given free choice between the two. Completion of an additional 52 weeks of this study may provide a more definite conclusion regarding patient satisfaction with these two injection regimens.

## Supplementary Information

Below is the link to the electronic supplementary material.

Supplementary file1 (PDF 136 KB)

Supplementary file2 (PDF 136 KB)

## Data Availability

The data that support the findings of this study are available from the corresponding author upon reasonable request.
