# Supplementary material for: Randomized crossover comparison of two teriparatide self-injection regimens for primary osteoporosis: Interim report (end of 52-week treatment) of the Japanese Osteoporosis Intervention Trial 06 (JOINT-06)
Source: J Bone Miner Metab. 2025 Feb 18;43(3):284–92. doi: 10.1007/s00774-025-01586-y (PMC12089251; doi:10.1007/s00774-025-01586-y)
Supplement: Supplementary file 1 — Supplementary file1 (PDF 136 KB) [file 774_2025_1586_MOESM1_ESM.pdf]

Article title: Randomized crossover comparison of two teriparatide self-injection regimens for primary osteoporosis: Interim report (end of 52-week treatment) of the Japanese Osteoporosis Intervention Trial 06 (JOINT-06)

Journal name: *Journal of Bone and Mineral Metabolism*

Authors: Satoshi Soen, Yukari Uemura, Shiro Tanaka, Yasuhiro Takeuchi, Naoto Endo, Junichi Takada, Satoshi Ikeda, Jun Iwamoto, Nobukazu Okimoto, Sakae Tanaka

Corresponding author: Satoshi Soen

Affiliation: Soen Orthopaedics, Osteoporosis and Rheumatology Clinic, Kobe, Hyogo, Japan

Email: nra48207@nifty.com

Supplemental Table 1. Patients' baseline characteristics

Item		1/D-TPTD to 2/W-TPTD (n = 180)	2/W-TPTD to 1/D-TPTD (n = 178)	p
Age, years		75.9 ± 7.3	75.4 ± 6.9	0.511
Age at menopause, years		50.1 ± 4.8	49.8 ± 3.2	0.534
Height, cm		150.3 ± 6.1	150.1 ± 6.1	0.719
Body weight, kg		49.6 ± 7.7	50.0 ± 8.8	0.639
BMI, kg/m^2		22.0 ± 3.2	22.2 ± 3.7	0.556
BMD, T-score				
Lumbar spine (L2–L4)		−2.27 ± 1.80	−2.48 ± 1.29	0.284
Femoral neck		−3.29 ± 0.79	−3.22 ± 0.91	0.454
Total hip		−2.54 ± 0.88	−2.58 ± 1.04	0.744
Pain VAS score		31.5 ± 29.8	28.6 ± 29.5	0.355
EQ-5D utility score		0.88 ± 0.25	0.91 ± 0.22	0.200
Clinical fracture within 1 month	Yes	56 (31.1)	57 (32.0)	0.910
	No	124 (68.9)	121 (68.0)	
History of hip fracture	Yes	15 (8.3)	16 (9.0)	0.853
	No	165 (91.7)	162 (91.0)	
Complications	Yes	99 (55.0)	92 (51.7)	0.596
	No	81 (45.0)	86 (48.3)	
Previous treatment for osteoporosis	Yes	65 (36.1)	79 (44.4)	0.131
	No	115 (63.9)	99 (55.6)	

Data are presented as mean ± standard deviation or n (%).

1/D-TPTD, daily injection of teriparatide; 2/W-TPTD, twice-weekly injection of teriparatide; BMI, body mass index; BMD, bone mineral density; VAS, visual analogue scale; EQ-5D, EuroQoL-5 Dimension scale
