# Supplementary material for: Randomized crossover comparison of two teriparatide self-injection regimens for primary osteoporosis: Interim report (end of 52-week treatment) of the Japanese Osteoporosis Intervention Trial 06 (JOINT-06)
Source: J Bone Miner Metab. 2025 Feb 18;43(3):284–92. doi: 10.1007/s00774-025-01586-y (PMC12089251; doi:10.1007/s00774-025-01586-y)
Supplement: Supplementary file 2 — Supplementary file2 (PDF 136 KB) [file 774_2025_1586_MOESM2_ESM.pdf]

Article title: Randomized crossover comparison of two teriparatide self-injection regimens for primary osteoporosis: Interim report (end of 52-week treatment) of the Japanese Osteoporosis Intervention Trial 06 (JOINT-06)

Journal name: *Journal of Bone and Mineral Metabolism*

Authors: Satoshi Soen, Yukari Uemura, Shiro Tanaka, Yasuhiro Takeuchi, Naoto Endo, Junichi Takada, Satoshi Ikeda, Jun Iwamoto, Nobukazu Okimoto, Sakae Tanaka

Corresponding author: Satoshi Soen

Affiliation: Soen Orthopaedics, Osteoporosis and Rheumatology Clinic, Kobe, Hyogo, Japan

Email: nra48207@nifty.com

Supplemental Table 2. AEs reported during the study period

		1/D-TPTD (n = 310)		2/W-TPTD (n = 324)	
Incidence		13 (4.2%)		22 (6.8%)	
		Serious	Not serious	Serious	Not serious
Total AEs			14		35
	Chills	0 (0.0)	0 (0.0)	0 (0.0)	2 (0.6)
	Nausea	0 (0.0)	5 (1.6)	0 (0.0)	11 (3.4)
	Feeling abnormal	0 (0.0)	2 (0.6)	0 (0.0)	2 (0.6)
	Gastrointestinal disorder	0 (0.0)	0 (0.0)	0 (0.0)	1 (0.3)
	Diarrhea	0 (0.0)	1 (0.3)	0 (0.0)	1 (0.3)
	Arthralgia	0 (0.0)	0 (0.0)	0 (0.0)	1 (0.3)
	Increased blood calcium	0 (0.0)	1 (0.3)	0 (0.0)	0 (0.0)
	Malaise	0 (0.0)	0 (0.0)	0 (0.0)	1 (0.3)
	Decreased appetite	0 (0.0)	2 (0.6)	0 (0.0)	2 (0.6)
	Spinal compression fracture	0 (0.0)	1 (0.3)	0 (0.0)	0 (0.0)
	Hypotension	0 (0.0)	0 (0.0)	0 (0.0)	1 (0.3)
	Headache	0 (0.0)	0 (0.0)	0 (0.0)	4 (1.2)
	Palpitations	0 (0.0)	0 (0.0)	0 (0.0)	1 (0.3)
	Dizziness	0 (0.0)	0 (0.0)	0 (0.0)	2 (0.6)
	Abdominal pain	0 (0.0)	0 (0.0)	0 (0.0)	1 (0.3)
	Abdominal discomfort	0 (0.0)	0 (0.0)	0 (0.0)	1 (0.3)
	Drug eruption	0 (0.0)	2 (0.6)	0 (0.0)	0 (0.0)
	Vomiting	0 (0.0)	0 (0.0)	0 (0.0)	2 (0.6)

Data are presented as n (%).

AEs, adverse events; 1/D-TPTD, daily injection of teriparatide; 2/W-TPTD, twice-weekly injection of teriparatide
